# Recommendations on cutaneous and hematological manifestations of Sjögren’s disease by the Brazilian Society of Rheumatology

**DOI:** 10.1186/s42358-024-00391-x

**Published:** 2024-07-09

**Authors:** Alisson Pugliesi, Danielle Christinne Soares do Egypto, Guilherme Duffles, Diego Ustárroz Cantali, Sandra Gofinet Pasoto, Fabiola Reis Oliveira, Valeria Valim, Maria Lucia Lemos Lopes, Samira Tatiyama Miyamoto, Marilena Leal Mesquita Silvestre Fernandes, Sonia Cristina de Magalhães Souza Fialho, Aysa César Pinheiro, Laura Caldas dos Santos, Simone Appenzeller, Sandra Lucia Euzébio Ribeiro, Tatiana Nayara Libório-Kimura, Maria Carmen Lopes Ferreira Silva Santos, Juliana D´Agostino Gennari, Roberta Pernanbuco, Karina Gatz Capobiano, Vinicius Tassoni Civile, Ana Carolina Pereira Nunes Pinto, César Ramos Rocha-Filho, Aline Pereira da Rocha, Virginia Fernandes Moça Trevisani

**Affiliations:** 1 Disciplina de Reumatologia, Departamento de Ortopedia, Reumatologia e Traumatologia da Faculdade de Ciências Médicas da Universidade Estadual de Campinas (UNICAMP), R. Tessália Vieira de Camargo, 126 - Cidade Universitária, Campinas, SP CEP: 13083-887 Brazil; 2 Disciplina de Reumatologia, Departamento de Medicina Interna, Centro de Ciências Médicas, Universidade Federal da Paraíba (UFPB), Campus I– Lot, Cidade Universitária, Paraíba, PB CEP: 58051-900 Brazil; 3 Departamento de Hematologia e Hemoterapia da Universidade Estadual de Campinas (UNICAMP), R. Tessália Vieira de Camargo, 126 - Cidade Universitária, Campinas, SP CEP: 13083-887 Brazil; 4 Serviço de Reumatologia, Hospital São Lucas da Pontifícia Universidade Católica do Rio Grande de Sul (PUCRS), Av. Ipiranga, 6690 - Jardim Botânico, Porto Alegre, RS CEP: 90610-000 Brazil; 5 Disciplina de Reumatologia, Laboratório de Autoimunidade (DLC + LIM17), Hospital das Clinicas HCFMUSP, Faculdade de Medicina, Universidade de São Paulo, R. Dr. Ovídio Pires de Campos, 225– Cerqueira César, São Paulo, SP CEP: 05403-010 Brazil; 6 Hospital das Clínicas da Faculdade de Medicina de Ribeirão Preto (HCFMRP-USP), Av. Bandeirantes, 3900, Vila Monte Alegre, Ribeirão Preto, SP CEP: 14049-900 Brazil; 7 Serviço de Reumatologia, Hospital Universitário Cassiano Antônio de Moraes, Universidade Federal do Espírito Santo (UFES), Av. Marechal Campos, 1468, Maruípe, Vitória, ES CEP: 29075-910 Brazil; 8 Disciplina de Especialidades Clínicas, Departamento de Clínica Médica, Universidade Federal de Ciências da Saúde de Porto Alegre (UFCSPA), R. Sarmento Leite, 245 - Centro Histórico de Porto Alegre, Porto Alegre, RS CEP: 90050-170 Brazil; 9 Departamento de Educação Integrada em Saúde, Universidade Federal do Espírito Santo (UFES), Av. Marechal Campos, 1468, Maruípe, Vitória, ES CEP: 29040-090 Brazil; 10 Serviço de Reumatologia, Hospital Federal dos Servidores do Estado do Rio de Janeiro, R. Sacadura Cabral, 178, Saúde, Rio de Janeiro, RJ CEP: 20221-903 Brazil; 11 Centro Catarinense de Imunoterapia, R. Santos Dumont, 182– Centro, Florianópolis, SC CEP: 88015-020 Brazil; 12 Serviço de Reumatologia, Universidade Federal de Pernambuco (UFPE), Av. Prof. Moraes Rego, 1235, Cidade Universitária, Recife, PE CEP: 50670-901 Brazil; 13 Departamento de Oftalmologia, Escola Paulista de Medicina– Universidade Federal de São Paulo (EPM-UNIFESP), Rua Botucatu, 820, Vila Clementino, São Paulo, SP CEP: 04023-062 Brazil; 14 Disciplina de Reumatologia, Universidade Federal do Amazonas, Rua Afonso Pena, 1053, Manaus, AM CEP: 69020-160 Brazil; 15 Departamento de Patologia e Medicina Legal, Universidade Federal do Amazonas, Rua Afonso Pena, 1053, Manaus, AM CEP: 69020-160 Brazil; 16 Departamento de Patologia, Hospital Universitário Cassiano Antônio de Moraes, Universidade Federal do Espírito Santo (UFES), Av. Marechal Campos, 1468, Maruípe, Vitória, ES CEP: 29075-910 Brazil; 17 Serviço de Reumatologia da Santa Casa de São Paulo, R. Dr. Cesário Mota Júnior, 112, Vila Buarque, São Paulo, SP CEP: 01221-020 Brazil; 18 Serviço de Reumatologia do Hospital do Servidor público do estado de São Paulo (HSPE- IAMSPE), Rua Pedro de Toledo 1800, São Paulo, SP CEP 04039-000 Brazil; 19 Serviço de Reumatologia do Hospital Moinhos de Vento - Porto Alegre, Rua Ramiro Barcelos 910, Porto Alegre, RS CEP: 90035-000 Brazil; 20 Disciplina de Medicina de Urgência e Medicina Baseada em Evidências, Escola Paulista de Medicina– Pós-Graduação em Saúde Baseada em Evidências, Universidade Federal de São Paulo (EPM-UNIFESP), Rua Botucatu, 740, Vila Clementino, São Paulo, SP CEP: 04023-062 Brazil; 21 Centro Cochrane do Brasil Universidade Federal de São Paulo (EPM-UNIFESP), Rua Botucatu, 740, Vila Clementino, São Paulo, SP CEP: 04023-062 Brazil; 22 Universidade Paulista, Rua Vergueiro, 1211, Paraíso, São Paulo, SP CEP: 01504-001 Brazil; 23 Centro Cochrane Iberoamericano, Hospital de la Santa Creu i Sant Pau, C/ de Sant Quintí, 89, Horta-Guinardó, Barcelona, 08025 Spain; 24 Disciplina de Reumatologia, Universidade de Santo Amaro (UNISA), Rua Enéas Siqueira Neto, Jardim das Imbuias, São Paulo, SP CEP: 04829-300 Brazil

## Abstract

Sjogren’s disease (SjD) is an autoimmune disease that is characterized not only by the sicca symptoms it causes but also by its systemic nature, which is capable of several and not yet fully understood extraglandular manifestations. To gain a clearer understanding of these manifestations as well as a better practical approach, a panel of experts from the Brazilian Society of Rheumatology conducted a systematic review and meta-analysis on the identification of epidemiologic and clinical features of the extraglandular manifestations present in ESSDAI (EULAR Sjogren´s syndrome disease activity index), followed by a voting panel with recommendations for clinical practice. This publication is complementary to others already published and covers cutaneous and hematological manifestations, with prevalence data generated by a meta-analysis of 13 clinical or laboratory manifestations and 6 clinical management recommendations.

**Supplementary Information:**

The online version contains supplementary material available at 10.1186/s42358-024-00391-x.

## Background

Sjogren’s disease (SjD) is a systemic autoimmune disease, marked not only by the sicca symptoms it causes but also by its systemic nature, which is capable of various extraglandular manifestations [1, 2]. With an estimated worldwide prevalence of 0.01–0.05% [3, 4], its pathogenesis begins with glandular dysfunction resulting from the infiltration of T lymphocytes. This is followed by the activation of B lymphocytes and synthesis of autoantibodies capable of immune manifestations at a distance from the glandular structures [5].

Systemic extraglandular manifestations occur in around 40–50% of patients and can be severe. They include fatigue; myalgia; arthralgia and/or arthritis; gastrointestinal manifestations; and vascular, renal, pulmonary, neurological, cutaneous, or hematologic involvement. The severity of systemic involvement can be assessed using the ESSDAI (EULAR Sjögren’s syndrome disease activity index). In this tool, each manifestation mentioned corresponds to a domain. When systemic involvement is present, the patient receives a score that can vary from 0 to 2, 0 to 3, or 0 to 5 (depending on the domain). The sum of the affected domains generates the final ESSDAI value [6].

Despite being extensively validated and recommended for grading systemic involvement, the ESSDAI does not propose a routine diagnostic assessment for patients with SjD, which is an unmet need for physicians caring for patients with SjD. In addition, there are limited divergent data on the prevalence of systemic manifestations.

To address these gaps, the Sjögren’s Disease Committee of the Brazilian Society of Rheumatology conducted a broad systematic review of the literature on studies investigating extraglandular symptoms in Sjögren’s patients. The committee then gathered experts in the field and published recommendations for the screening and management of SjD patients with articular, pulmonary, renal, hepatic, gastrointestinal, and pancreatic manifestations [7, 8]. The current study represents an effort by the Brazilian Society of Rheumatology with the objective of retrieving the best available evidence and providing guidance for the identification of symptoms, diagnosis, monitoring, prognosis, and treatment of cutaneous and hematologic manifestations of SjD.

## Methods

We conducted a systematic review of the diagnosis and prevalence of cutaneous and hematological manifestations in patients diagnosed with SjD according to the 2002, 2012, and 2016 classification criteria [9–11], following the recommendations proposed by the Cochrane Collaboration Handbook [12]. Questions were asked about the diagnosis and prevalence of cutaneous and hematological manifestations of SjD. An individualized search strategy for the different systemic manifestations was performed (Supplementary Material) for the Cochrane Central, MEDLINE, Embase, and LILACS databases.

The strategy was conducted with no restrictions on language or publication date. Only publications involving patients with the primary form of the disease (no other connective tissue disease) and those older than 18 years of age were selected. Observational studies in which the primary research question concerned the diagnosis and prevalence of individualized systemic manifestations were included. Articles that were duplicates across different databases were excluded along with those that did not concentrate on diagnosis and prevalence. To assess the diagnosis of systemic manifestations, preference was given to diagnostic accuracy studies. In the absence of such studies, we included any observational study that reported the use of diagnostic tests for detecting systemic manifestations of SjD.

The first study selection stage consisted of reading the titles and encompassing abstracts and keywords. The titles and abstracts of articles identified by the search strategy were independently assessed by two authors of the present study (AP and DCSE). In case of disagreement, a third author decided (VFMT). In the second stage, the investigators independently evaluated the full text of the articles and selected them according to pre-specified eligibility criteria.

To estimate the prevalence of cutaneous and hematological manifestations, a meta-analysis was carried out on the selected studies. We considered studies that specified both the number of patients affected by cutaneous or hematological manifestations and the total number of patients with SjD included. The statistical heterogeneity observed in these meta-analyses was anticipated by our review group, as it is a common occurrence in meta-analyses of prevalence. This expectation was due to the extensive range of patient characteristics, various available classification criteria (2002, 2012, and 2016), and different methods used to evaluate systemic manifestations. The risk of bias was evaluated using the Joanna Briggs Institute Prevalence Critical Appraisal Tool [13] (Supplementary Material). For the meta-analysis, we pooled clinical data by extracting the number of events and total number of patients to conduct a proportion meta-analysis. To estimate the overall proportion and present the pooled results with their respective 95% confidence intervals (CI), we employed a generalized linear mixed model (GLMM) method with a random-effects model. Calculations were performed using logit transformation in the “meta” and “metafor” packages from R software (version 3.6.1). Based on the data from the systematic review and meta-analysis, recommendations were formulated by Rheumatologists from the Sjögren´s Disease Committee of the Brazilian Society of Rheumatology. Agreement on these recommendations was achieved through online and in-person meetings using the Delphi Method [8].

## Results

**Fig. 1:**
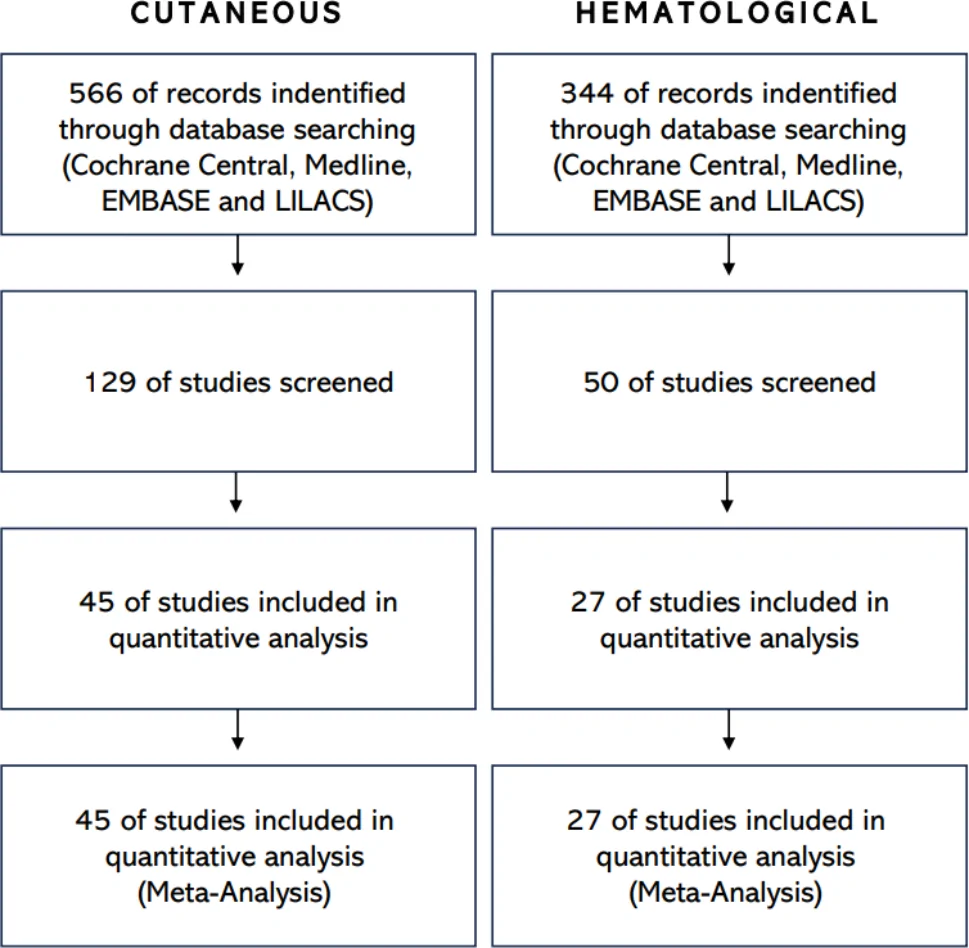
Flowchart of the studies selected for meta-analysis

Figure 1 summarizes the steps involved in this systematic review. Important topics were described in sections reserved for cutaneous and hematologic manifestations, and based on these, two recommendations for cutaneous diseases and four recommendations for hematologic diseases were made (Table 1). Forest plots of the prevalence meta-analysis are shown at the end of each manifestation topic.

**Table 1:** Recommendations for the evaluation of cutaneous and hematological manifestations of Sjögren’s disease

1. Cutaneous vasculitis (palpable or nonpalpable purpura in the lower extremities and urticarial vasculitis) is associated with a higher risk of developing other extraglandular manifestations and lymphoma, especially in the presence of cryoglobulinemia and hypocomplementemia. In such cases, follow-up at smaller, regular intervals is recommended. (100% of agreement; Strength of Recommendation: Strong)
2. A biopsy can be performed for the differential diagnosis of skin manifestations. However, when the skin lesion is purpura, vasculitis can be clinically diagnosed. (93.4% of agreement; Strength of Recommendation: Strong)
3. Blood counts should be assessed at least every 3–6 months (or earlier if necessary) in patients with SjD, since manifestations such as anemia, thrombocytopenia, and leukopenia (neutropenia and lymphopenia) are common. Before being attributed to disease activity, a broad evaluation of other causes should be performed. (100% agreement; Strength of Recommendation: Strong)
4. Complement C3 and C4 fractions and protein electrophoresis should be performed regularly as an additional step in assessing disease activity. (100% agreement; Strength of Recommendation: Strong)
5. Cryoglobulins should be measured at least once during follow-up (and regularly if the result is positive) and in the presence of manifestations suggestive of vasculitis (mainly peripheral neuropathy, glomerulonephritis, and purpura). (93.4% agreement; Strength of Recommendation: Conditional).
6. A systematized evaluation of lymphoma signs and symptoms must be performed in every follow-up consultation, especially in those presenting with risk factors. It should involve directed anamnesis (fever, night sweats, weight loss) and physical examination (palpation of the spleen, lymph nodes, and salivary glands). The presence of any suggestive findings in this evaluation merits prompt assessment for lymphomatous transformation, with those related to enlargement of the parotid glands or other salivary glands (chronic and/or asymmetrical enlargement and/or hardening/adherence and/or refractory to clinical measures) being of particular importance, as they are the most common site of lymphoma in patients with Sjögren’s. (100% agreement; Strength of Recommendation: Strong).

### Cutaneous manifestations

Skin involvement in SjD is relatively common and occurs in many patients. Several manifestations have been identified that significantly reduce patients’ quality of life by causing irritating symptoms, such as itching and pain. Table 2 summarizes the key points of the skin manifestations found in our systematic review.

**Table 2:** Key points of cutaneous manifestations in SjD

• Diverse skin manifestations can occur in SjD, such as xeroderma, eyelid dermatitis, annular erythema or subacute cutaneous lupus erythematosus (SCLE), angular cheilitis, Raynaud’s phenomenon, vasculitis, livedo reticularis, urticaria, panniculitis, granuloma annulare, nodular localized cutaneous amyloidosis (NLCA), and lymphomatoid papulosis (LyP)
• Skin lesions that indicate disease activity are erythema multiforme, SCLE, urticarial vasculitis, and purpura
• Purpura is a prognostic factor for systemic disease activity and lymphoma

The pathophysiological mechanism of mucocutaneous lesions in SjD is not fully understood, and an inflammatory response is usually identified [14, 15].

The classification of skin activity in ESSDAI [6] is based on the presence of certain types of skin lesions: absence of active skin lesions, 0 (no activity); erythema multiforme, 1 (low activity); limited cutaneous vasculitis, including urticarial vasculitis, purpura limited to feet and ankle, or subacute acute lupus erythematous (SCLE) = 2 (moderate activity); and diffuse cutaneous vasculitis, including urticarial vasculitis, diffuse purpura, or vasculitis-related ulcers = 3 (high activity).

The skin manifestation with the greatest clinical and prognostic importance associated with SjD is vasculitis, and patients may present with a wide variety of non-vasculitic lesions. Among the most common dermatological manifestations in SjD, we identified xeroderma, eyelid dermatitis, annular erythema, angular cheilitis, Raynaud’s phenomenon, and vasculitis, including nonpalpable purpura, livedo reticularis, urticaria, panniculitis, granuloma annulare, nodular localized cutaneous amyloidosis (NLCA), and lymphomatoid papules (LyP) [14, 16].

**Fig. 2:**
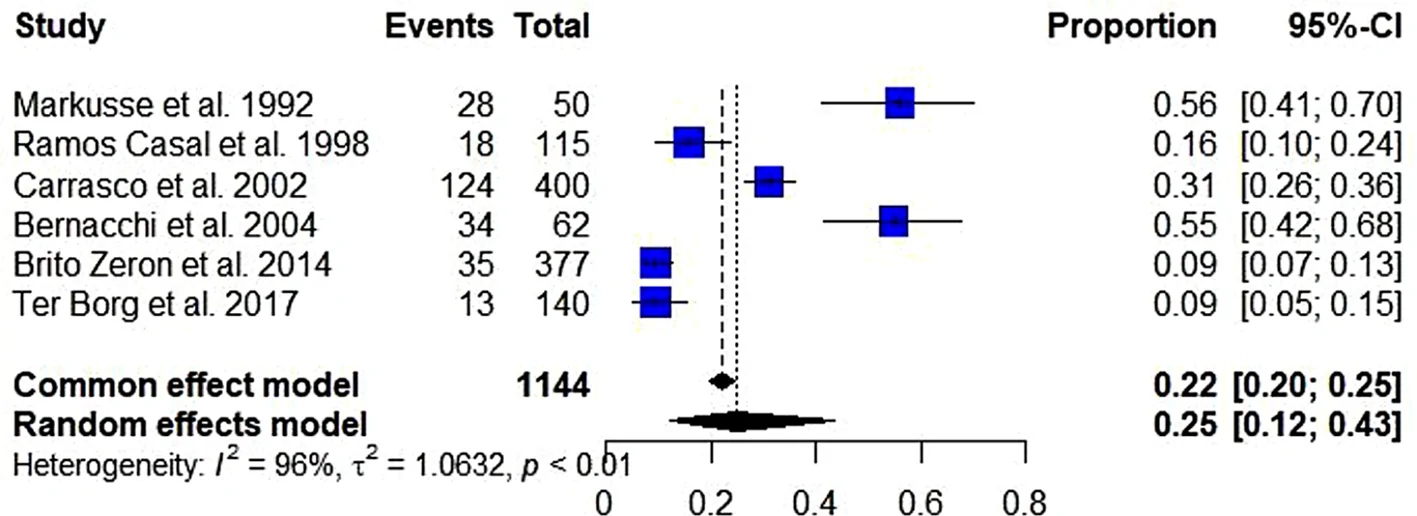
Meta-analysis of xeroderma prevalence in Sjögren’s disease

Xeroderma is a common condition characterized by dry scaly skin with reduced elasticity, usually causing itching and burning. This is the most frequent cutaneous manifestation of SjD, with studies showing its occurrence in 23–67% of cases [17–19]. The etiology of xeroderma is not fully understood. It was thought to be secondary to a reduction in sweat due to chronic inflammation of the exocrine glands; however, Bernacchi et al. did not report chronic inflammatory atrophy of sweat glands or reduced sweat rate, indicating that it was secondary to changes in the biochemical properties of the epidermis (increased epidermal proliferation and disrupted epidermal differentiation) [18, 20, 21]. Patients with xeroderma often develop rash complications with scratches on the skin and secondary hyperpigmentation [14, 17, 22]. In our meta-analysis, we identified a prevalence of 25% (95% CI 12–43%; 6 studies; 1144 participants) for xeroderma (Fig. 2).

**Fig. 3:**
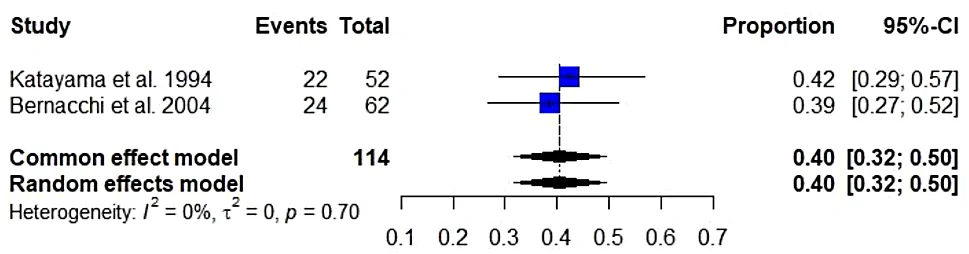
Meta-analysis of the prevalence of eyelid dermatitis in SjD patients

Eyelid dermatitis is not a specific cutaneous manifestation of SjD and has been identified in approximately 40% of patients [23, 24]. It is often secondary to eye rubbing due to burning and itching caused by xerophthalmia [17, 24]. We identified a prevalence of 40% (95% CI 32–50%; two studies; 114 participants) of eyelid dermatitis (Fig. 3).

**Fig. 4:**
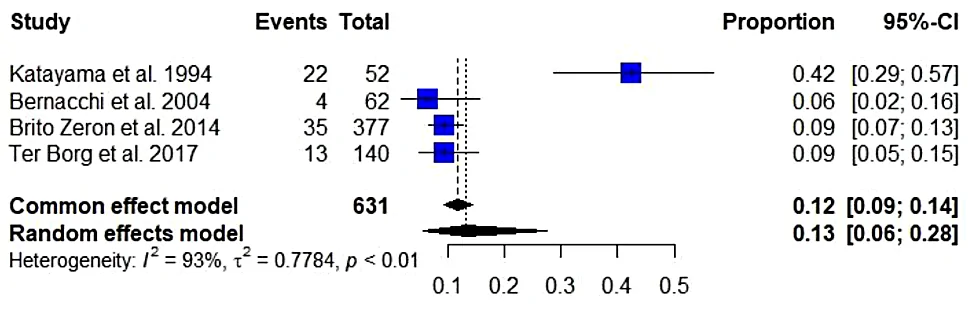
Meta-analysis of the prevalence of annular erythema in SjD patients

Annular erythema (AE) preferentially affects Asians and is rare in Caucasian patients. It is considered equivalent to SCLE lesions in whites [25, 26]. The first case of AE in a non-Asian population was described in 2014. It presents with lesions similar to erythema multiforme (erythematous lesions), often photosensitive, with a high border and clear center [27, 28]. Histopathology showed perivascular lymphocytic infiltration in 100% of the cases. AE occur more frequently in photo-exposed areas or in a disseminated form; however, the face is usually spared. It is strongly associated with positivity for anti-Ro/SSA and anti-La/SSB antibodies [27, 29]. AE is included in the cutaneous domain of the ESSDAI [6] and is classified as a manifestation of moderate activity [6, 30, 31]. In our Meta-analysis, we found a prevalence of 13% (95% CI 6–28%; 4 studies; 631 participants) (Fig. 4).

Angular cheilitis manifests itself as erythema with cracks and painful or painless sores in the corners of the mouth, secondary to anemia, or as a fungal or bacterial infection. It was identified as the second most frequent oral mucosal lesion in patients with SjD [32].

Raynaud’s phenomenon occurs in 20 to 35% of SjD patients, with a high incidence in those with cutaneous vasculitis [23].

#### Recommendation 1

- Cutaneous vasculitis (palpable or nonpalpable purpura in the lower extremities and urticarial vasculitis) is associated with a higher risk of developing other extraglandular manifestations and lymphoma, especially in the presence of cryoglobulinemia and hypocomplementemia. In such cases, follow-up at smaller, regular intervals is recommended. (100% of agreement; Strength of Recommendation: Strong)

#### Recommendation 2

- A biopsy can be performed for the differential diagnosis of skin manifestations. However, when the skin lesion is purpura, vasculitis can be clinically diagnosed. (93.4% of agreement; Strength of Recommendation: Strong)

**Fig. 5:**
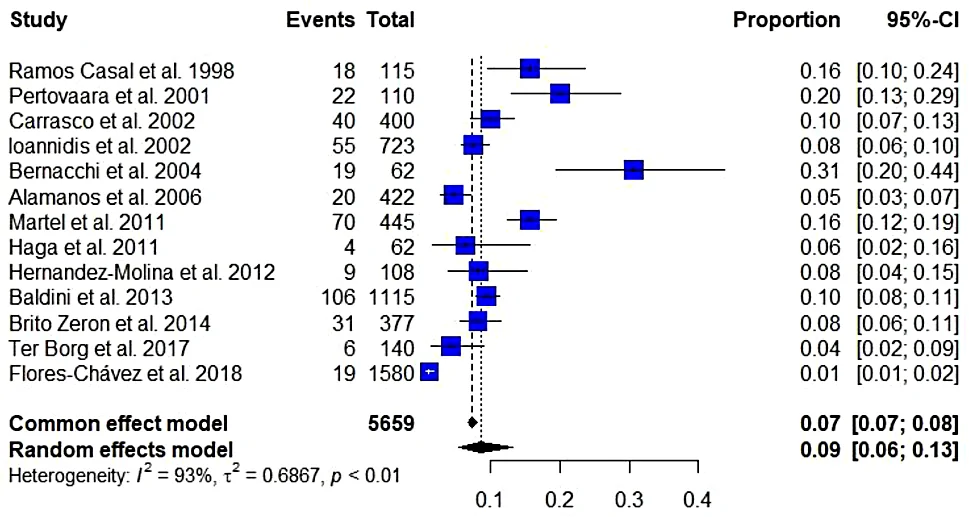
Meta-analysis of the prevalence of cutaneous vasculitis in SjD

Cutaneous vasculitis (CV) is the most important extraglandular manifestation in terms of prognosis and is associated with greater disease severity and systemic comorbidities [17]. It has been reported in up to 30% of SjD cases, and patients may present with a single episode or recurrent course [23, 33]. In our meta-analysis, we found a prevalence of 9% (95% CI 6–13%; 13 studies; 5659 participants) of this manifestation (Fig. 5).

The most common clinical presentation is palpable or non-palpable lower-extremity purpura [33], followed by urticarial vasculitis. Less frequent lesions include erythematous macules, papules, ulcers, and ischemia [34]. CV predominantly affects small vessels, with rare reports of medium-vessel involvement. Histopathology shows leukocytoclastic vasculitis in 90% of cases; however, skin biopsy is not mandatory for the diagnosis of vasculitis associated with SjD [17, 23, 33–36]. Ramos-Casals et al found an association between low C4 levels and a high incidence of cutaneous vasculitis [37].

Waldenström’s hypergammaglobulinemia is a relatively common vasculitic manifestation in SjD, characterized by palpable purpura, marked polyclonal hypergammaglobulinemia, and high levels of rheumatoid factor and anti-Ro/anti-La antibodies [22, 33]. This condition is not usually associated with severe systemic disease but with peripheral neuropathy [38].

The presence of cryoglobulinemia with palpable purpura is associated with a higher prevalence of extraglandular disease and lymphoma, with high morbidity and mortality rates [37, 39, 40]. Cryoglobulinemia is associated with leukocytoclastic vasculitis in SjD in approximately 30% of the cases [34].

Laboratory markers of disease severity (low C3/C4 levels, positive rheumatoid factor, hypergammaglobulinemia, and cryoglobulins) were more frequent in patients with cutaneous vasculitis [34, 37].

The ESSDAI [6] classified the presence of vasculitis in SjD according to the extent of skin involvement (moderate activity when <18% and high activity when >18% of body surface area is involved) and the presence of ulcers (high activity).

There are reports of NLCA in SjD [41]. Other cutaneous lymphoproliferative disorders associated with SjD include LyP and cutaneous lymphomas. LyP is a benign condition in which 5–10% of cases progress to malignant lymphoma [42]. Other skin manifestations of SjD include vitiligo, alopecia, erosive lichen planus, erythema multiforme-like and erythema nodosum-like lesions, Sweet’s syndrome, granulomatous panniculitis, and subcorneal pustular dermatosis. However, these findings have been isolated and have unclear significance [22, 43, 44].

### Hematological manifestations

For hematological manifestations, we will address the ESSDAI domain of the same name and the so-called biological domain (relating to laboratory alterations of the complement system, cryoglobulin positivity, and alterations in gamma globulins) since they intersect with hematology. Table 3 summarizes the key points of hematological manifestations found in our systematic review.

**Table 3:** Key points of hematological manifestations in SjD

• Anemia, lymphopenia, and neutropenia are the most frequent manifestations
• Complement consumption and the presence of cryoglobulin are marker of disease activity and overall risk of mortality
• Persistent parotid enlargement, C4 consumption, cryoglobulinemia, rheumatoid factor, and disease activity are the main risk factors for lymphoma in SjD
• The main type of lymphoma found in SjD is the MALT (mucosa-associated lymphoid tissue) lymphoma of the salivary gland

#### Recommendation 3

- Blood counts should be assessed at least every 3–6 months (or earlier if necessary) in patients with SjD, since manifestations such as anemia, thrombocytopenia, and leukopenia (neutropenia and lymphopenia) are common. Before being attributed to disease activity, a broad evaluation of other causes should be performed. (100% agreement; Strength of Recommendation: Strong).

**Fig. 6:**
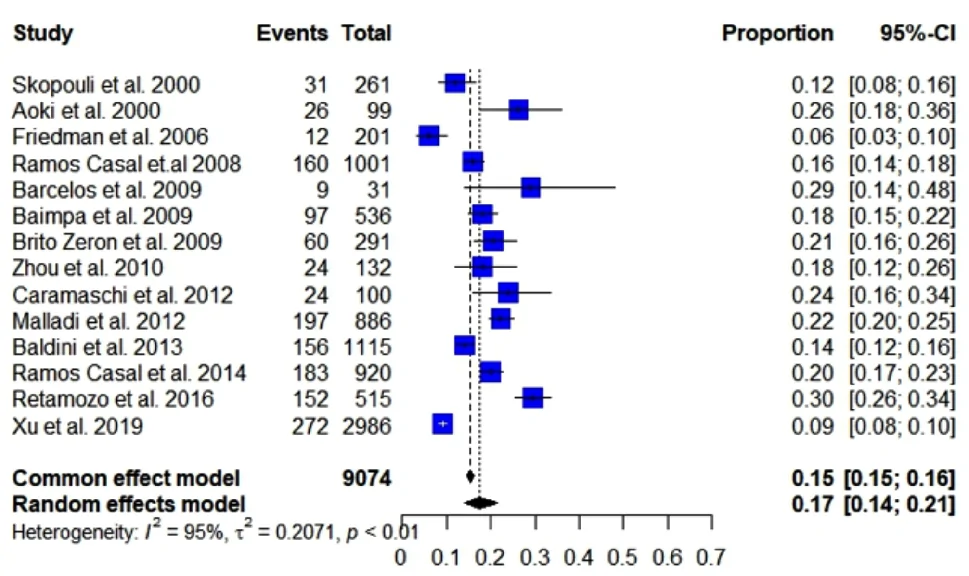
Meta-analysis of leukopenia in SjD

**Fig. 7:**
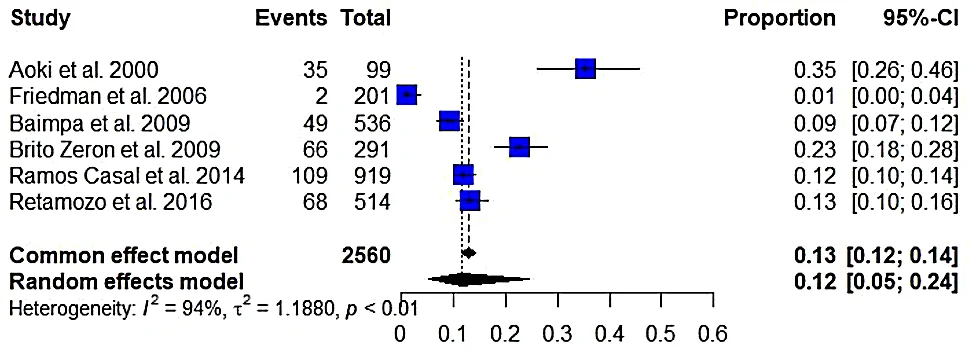
Meta-analysis of lymphopenia in SjD

**Fig. 8:**
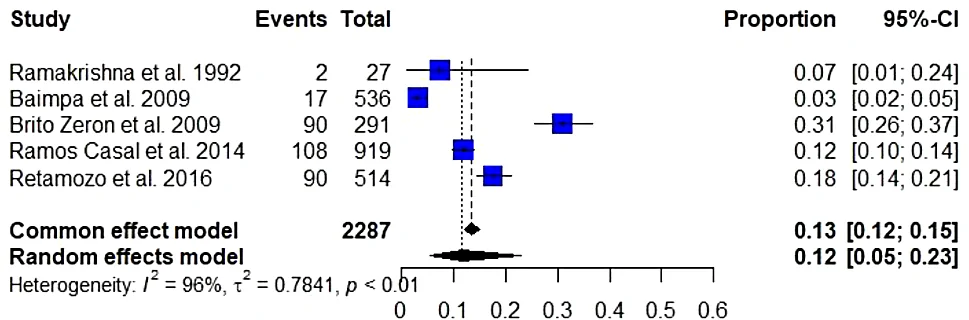
Meta-analysis of neutropenia in SjD

Leukopenia is one of the most important hematological manifestations [45, 46]. We have found a prevalence of leukopenia (<4000 cells/mm^3^), lymphopenia (<1500 cells/mm^3^), and neutropenia (<1500 cells/mm^3^) of 17% (95% CI 14 to 21%; 14 studies; 9074 participants), 12% (95% CI 5 to 24%; 6 studies; 2560 participants) and 12% (95% CI 5 to 23%; 5 studies; 2287 participants) respectively (Figs. 6, 7 and 8) [2, 45–58].

The most likely mechanism is autoimmunity. As in other contexts, neutropenia is associated with greater severity given the risk of bacterial infectious complications.

**Fig. 9:**
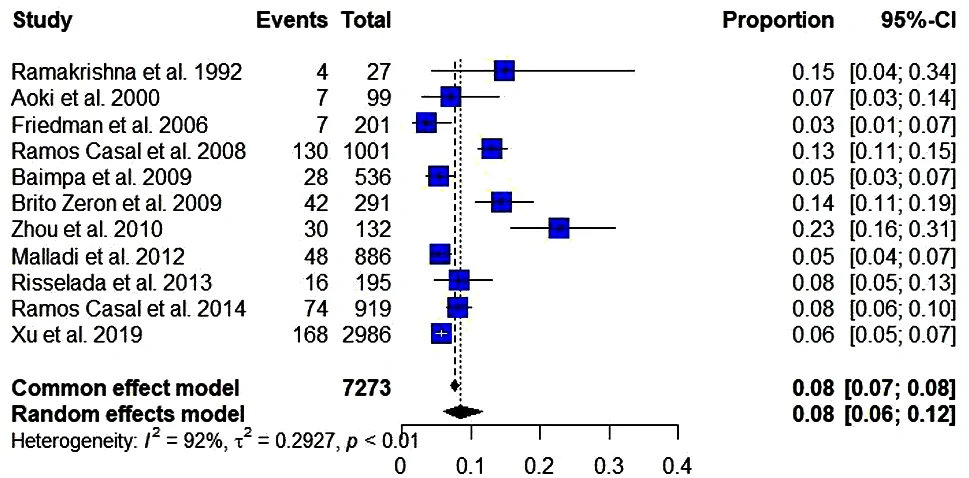
Meta-analysis of thrombocytopenia in SjD

Thrombocytopenia (<150,000 cells/mm^3^) was found in 3–14% of cases [46, 50], with an estimated prevalence of 8% (95% CI 6–12%; 11 studies; 7273 participants) in our meta-analysis (Fig. 9) [2, 45–47, 49, 50, 52, 53, 55, 57, 58]. A study evaluating the most frequent autoimmune etiologies of immune thrombocytopenia found SjD in 18.82% of cases, second only to systemic lupus erythematosus (SLE) [59].

**Fig. 10:**
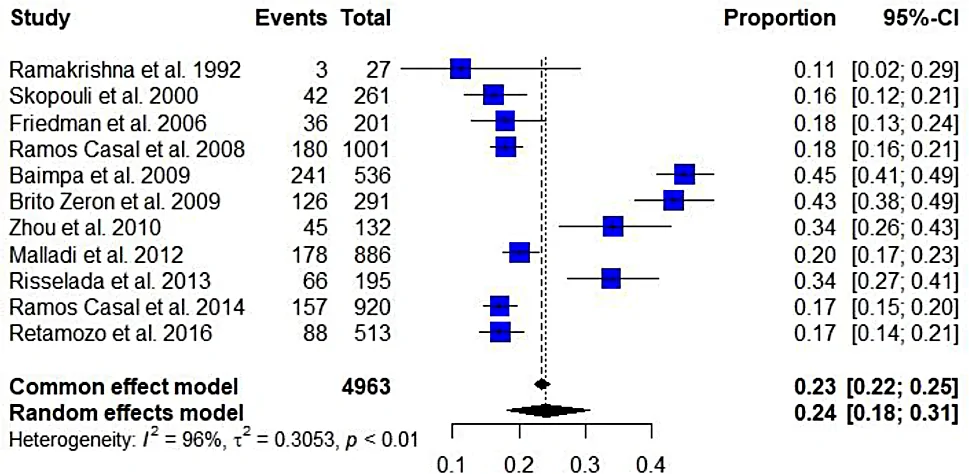
Meta-analysis of anemia in SjD

Anemia was the most prevalent manifestation, reaching 45% and with an estimated of 24% (95% CI 18 to 31%; 12 studies; 4963 participants in our meta-analysis (Fig. 10) [2, 45–47, 49, 50, 52, 53, 55, 56]. Zhou et al. reported that 69% of cases were classified as anemia of chronic disease, and 18% as autoimmune hemolytic anemia [53].

The ESSDAI presents a specific field in the hematological domain. In this field, only immune cytopenia were evaluated, and the score was given by the magnitude of the reduction in baseline values.

High activity is defined in the hematological field when hemoglobin, neutrophil, and platelet levels are below 8.0 g/dL, 500/mm^3^, and 50,000/mm^3^, respectively (one of these values is sufficient for the classification). There is no mention of lymphopenia in the criteria of high activity, as it presents a lower risk of complications than other cytopenias.

#### Recommendation 4

- Complement C3 and C4 fractions and protein electrophoresis should be performed regularly as an additional step in assessing disease activity. (100% agreement; Strength of Recommendation: Strong).

**Fig. 11:**
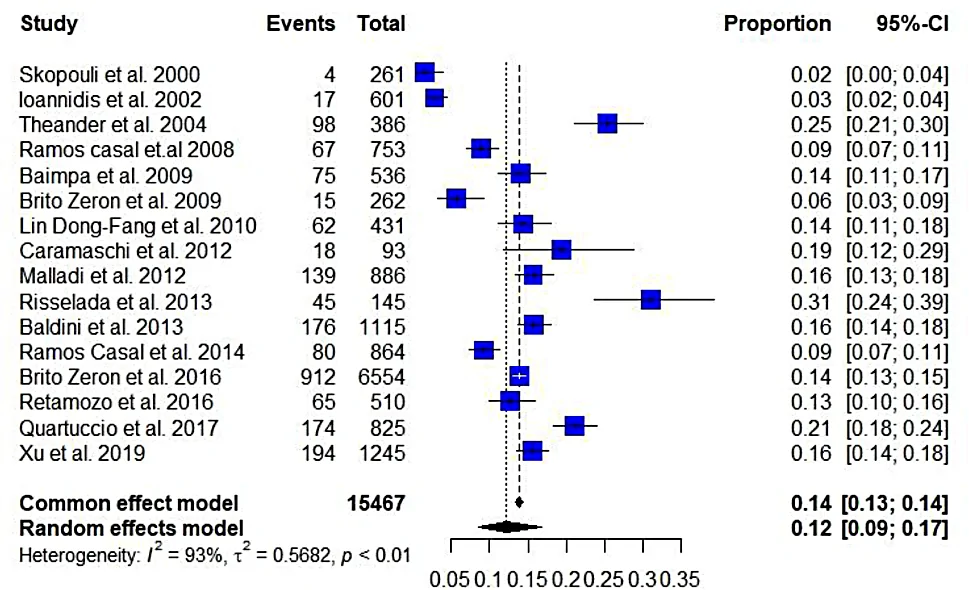
Meta-analysis of the prevalence of C3 consumption in SjD

**Fig. 12:**
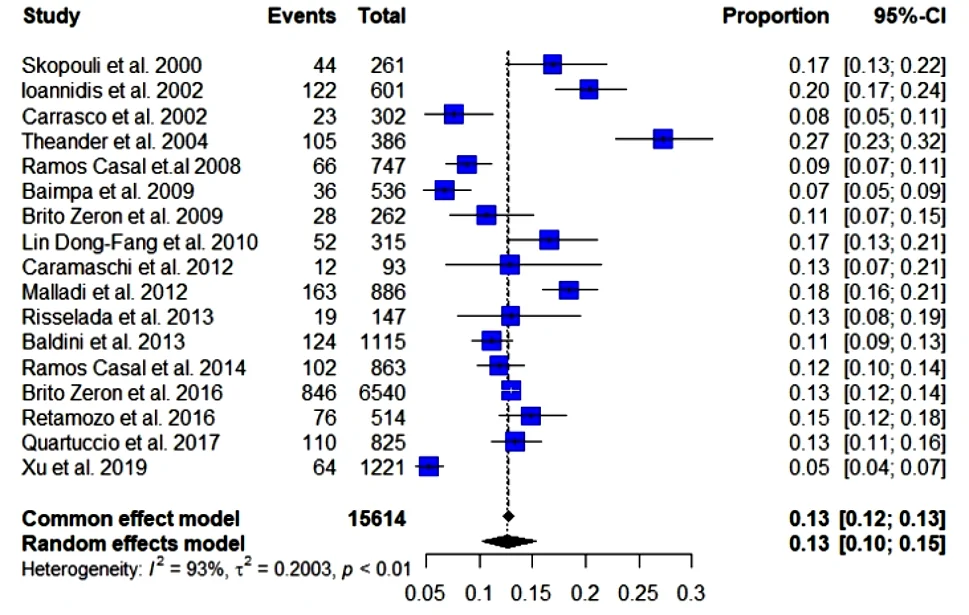
Meta-analysis of the prevalence of C4 consumption in SjD

The systematic review of the prevalence of complement consumption and serum cryoglobulin positivity also revealed clinically relevant data. When assessed in isolation, C3 consumption was present in 2–25% of cases [2, 60], and C4 consumption ranged from 5.0 to 37.2% [57, 60]. Our meta-analysis [2, 46–50, 52, 54–58, 60–64] found 12% (95% CI 9–17%; 16 studies; 15,467 participants) and 13% (95% CI 9–15%; 17 studies; 15,614 participants) of C3 and C4, respectively (Figs. 11 and 12).

Analysis from the Sjögren Big Data Project detected an association between complement consumption and a series of manifestations in other domains of ESSDAI (cutaneous, peripheral nervous system, and hematological) [63].

**Fig. 13:**
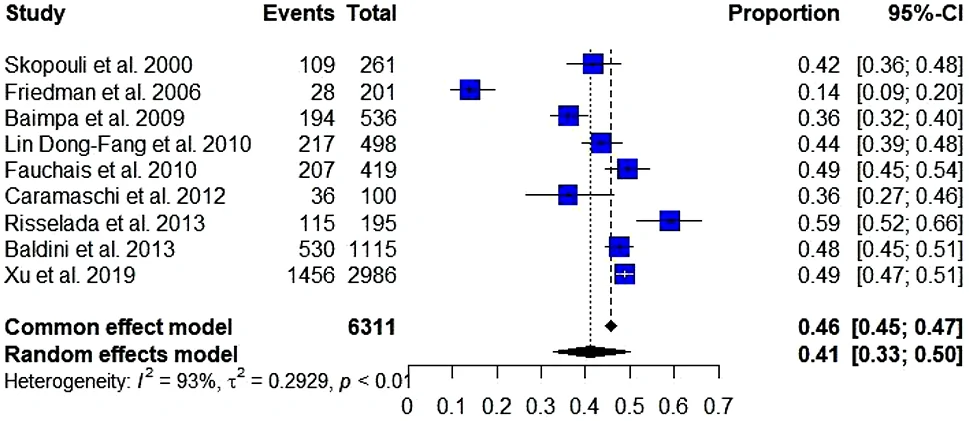
Meta-analysis of hypergammaglobulinemia prevalence in SjD

Regarding changes related to gamma globulins, the finding of hypergammaglobulinemia was frequent: 14 to 49% [57, 65, 66] with an estimated prevalence of 41% (95% CI 33 to 50%; 9 studies; 6311 participants) in our meta-analysis [2, 48, 50, 52, 54, 57, 64–66] (Fig. 13). In contrast, levels compatible with hypogammaglobulinemia showed much lower frequencies, varying from 2 to 6% [52, 66]. This study considered only disorders related to gamma globulin that were classified in a binary manner. Due to its diversity and low quantity, clonality characteristics were not evaluated in this systematic review.

The biological domain of ESSDAI includes gamma globulins and complements. There is no quantitative assessment of complement reduction; therefore, any decrease was classified as mild. Gammopathy disorders, however, can lead to the classification of mild or moderate activity.

#### Recommendation 5

- Cryoglobulins should be measured at least once during follow-up (and regularly if the result is positive) and in the presence of manifestations suggestive of vasculitis (mainly peripheral neuropathy, glomerulonephritis, and purpura). (93.4% agreement; Strength of Recommendation: Conditional).

**Fig. 14:**
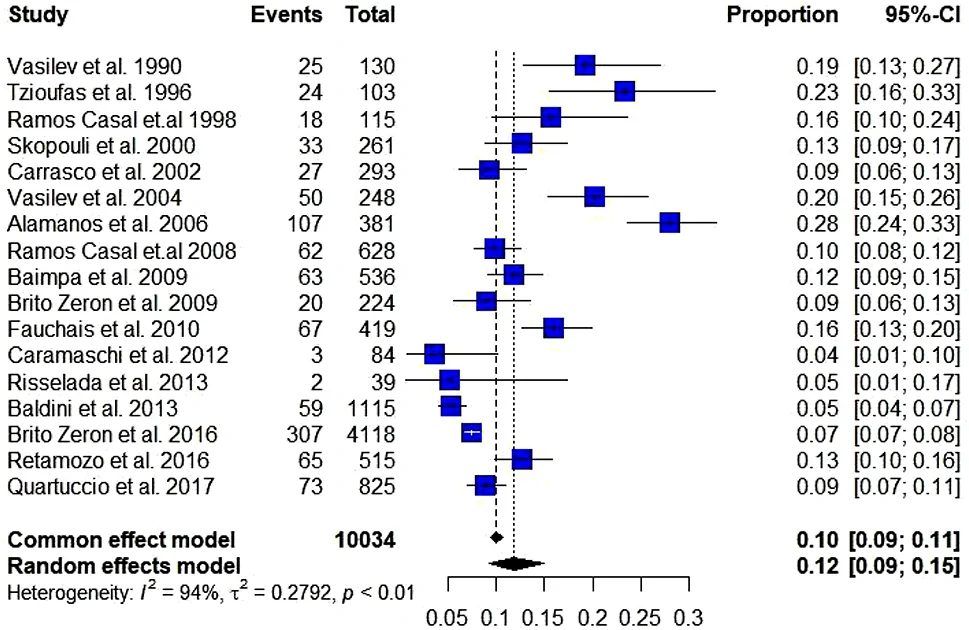
Meta-analysis of the prevalence of cryoglobulin in SjD

In the different studies, 3.6–28.0% of the patients were serum positive for cryoglobulin [54, 67] with an estimated prevalence of 12% (95% CI 9 to 15%; 17 studies; 10,034 participants) in our meta-analysis [2, 46–48, 52, 54, 56, 62, 63, 65, 67–71] (Fig. 14). The most common form of cryoglobulinemia in SjD is type 2 cryoglobulinemia. The positivity of cryoglobulinemia allows the classification of moderate activity in the biological domain of the ESSDAI, given the association of cryoglobulinemic vasculitis with higher overall mortality [56].

#### Recommendation 6

- A systematized evaluation of lymphoma signs and symptoms must be performed in every follow-up consultation, especially in those presenting with risk factors. It should involve directed anamnesis (fever, night sweats, weight loss) and physical examination (palpation of the spleen, lymph nodes, and salivary glands). The presence of any suggestive findings in this evaluation merits prompt assessment for lymphomatous transformation, with those related to enlargement of the parotid glands or other salivary glands (chronic and/or asymmetrical enlargement and/or hardening/adherence and/or refractory to clinical measures) being of particular importance, as they are the most common site of lymphoma in patients with Sjögren’s. (100% agreement; Strength of Recommendation: Strong).

The risk of lymphoma in the population with SjD is higher than that in the population without the disease, or even in those with RA and SLE [72]. Because of the risk of lymphoma, the mortality associated with SjD is lower than that of the general population [67]. Classified as an ESSDAI manifestation, the possibility of lymphomagenesis in patients with SjD and its relationship with other hematologic manifestations deserves emphasis in these recommendations.

The main type of lymphoma found in SjD is the MALT (mucosa-associated lymphoid tissue) lymphoma of the salivary gland. This is a B-lymphocyte lymphoma, non-Hodgkin’s lymphoma, classified as an extra-nodal marginal zone (ENMZ) lymphoma. Diffuse B-Cell Lymphoma (DBCL) and B lymphoma of the Nodal Marginal Zone (NMZ) are the second and third most common types of lymphoma, respectively [73].

A few steps have been hypothesized to explain the lymphomagenesis that occurs in SjD. Upon stimulation by cytokines and other substances, an increase in the pool of B lymphocytes in the glandular epithelial unit occurs. Oncogenes and tumor suppressor genes drive polyclonal B-lymphocytes to undergo a shift to monoclonality, the hallmark of lymphoma [74]. The transformation of a primary MALT lymphoma into a DBCL or NMZ could explain the high prevalence of these types in SJD [75, 76].

It is possible that there is an effect of the duration of the disease, since it takes an average of 6.5 years for lymphoma to appear, and that there is a cumulative risk as high as 18% in those with 20 years of disease [77]. Persistent parotid enlargement, C4 consumption, cryoglobulinemia, rheumatoid factor, and disease activity are often considered risk factors for lymphoma in SjD [74] and reflect the importance of the salivary gland epithelium and B lymphocytes in lymphomagenesis.

Persistent and/or asymmetric enlargement of the salivary glands (especially the parotid gland) is the main clinical finding that needs an evaluation directed towards the possibility of lymphoma [78]. In such cases, imaging evaluation and discussion with specialists are recommended for the evaluation of incisional or excisional biopsy. Fine needle aspiration is not recommended [79–81].

## Conclusion

In the third part of a series of systematic reviews and meta-analyses on the extraglandular manifestations of SjD conducted by the Brazilian Society of Rheumatology, prevalence data on cutaneous and hematological manifestations were generated, as well as six recommendations for the practical evaluation and diagnosis of these manifestations. The heterogeneity of the data found here points to the need for larger prospective epidemiological studies on the cutaneous and hematological manifestations of SjD, which will allow for a larger and more precise number of recommendations for future clinical practice.

## Electronic supplementary material

Below is the link to the electronic supplementary material.

Supplementary Material 1

## Data Availability

All data generated or analyzed during this study are included in this published article [and its Additional files].
