# Supplementary material for: Recommendations on cutaneous and hematological manifestations of Sjögren’s disease by the Brazilian Society of Rheumatology
Source: Adv Rheumatol. 2024 Jul 9;64:51. doi: 10.1186/s42358-024-00391-x (PMC13488840; doi:10.1186/s42358-024-00391-x)
Supplement: Supplementary file 1 [file 42358_2024_391_MOESM1_ESM.docx]

**Supplement material:**

1) **Search Strategy**

- **Cutaneous Manifestations**

**((("Sjogren's Syndrome"[Mesh] OR (Sjogrens Syndrome) OR (Syndrome, Sjogren's) OR (Sjogren Syndrome) OR (Sicca Syndrome) OR (Syndrome, Sicca))) AND ("Cutaneous Disease"[Mesh] OR (Disease, Cutaneous) OR (Diseases, Cutaneous) OR (Cutaneous Disease) OR (Blood Diseases) OR (Cutaneous Disease) OR (Disease, Cutaneous) OR (Diseases, Cutaneous) OR (Cutaneous Diseases) OR (Disease, Cutaneous) OR (Diseases, Cutaneous) OR (Cutaneous Disease))) AND (sensitiv*[Title/Abstract] OR sensitivity and specificity[MeSH Terms] OR (predictive[Title/Abstract] AND value*[Title/Abstract]) OR predictive value of tests[MeSH Term] OR accuracy*[Title/Abstract])**

- **Hematological Manifestations**

**((("Sjogren's Syndrome"[Mesh] OR (Sjogrens Syndrome) OR (Syndrome, Sjogren's) OR (Sjogren Syndrome) OR (Sicca Syndrome) OR (Syndrome, Sicca))) AND ("Hematologic Diseases"[Mesh] OR (Disease, Hematologic) OR (Diseases, Hematologic) OR (Hematologic Disease) OR (Blood Diseases) OR (Blood Disease) OR (Disease, Blood) OR (Diseases, Blood) OR (Hematological Diseases) OR (Disease, Hematological) OR (Diseases, Hematological) OR (Hematological Disease))) AND (sensitiv*[Title/Abstract] OR sensitivity and specificity[MeSH Terms] OR (predictive[Title/Abstract] AND value*[Title/Abstract]) OR predictive value of tests[MeSH Term] OR accuracy*[Title/Abstract])**

2) **Joanna Briggs Institute (JBI) Critical Appraisal Checklist**

**Cutaneous Manifestations**

| **Study ID** | **Was the sample frame appropriate to address the target**  **population?** | **Were study participants sampled in an appropriate way?** | **Was the sample size adequate?** | **Were the study subjects and the setting described in**  **detail?** | **Was the data analysis conducted with sufficient coverage**  **of the identified sample?** | **Were valid methods used for the identification of the**  **condition?** | **Was the condition measured in a standard, reliable way**  **for all participants?** | **Was there appropriate statistical analysis?** | **Was the response rate adequate, and if not, was the low**  **response rate managed appropriately?** |
| --- | --- | --- | --- | --- | --- | --- | --- | --- | --- |
| Alamanos et al. (2006) | + | + | + | + | - | - | - | + | + |
| Brito-Zerón et al. (2018) | + | + | + | + | + | + | - | + | + |
| Brito-Zerón et al. (2014) | + | - | - | + | + | + | + | + | - |
| Baldini et al. (2014) | + | - | - | + | - | + | + | + | - |
| Martel et al. (2011) | - | - | - | + | - | ? | ? | + | - |
| Xuan et al. (2017) | + | - | - | - | + | ? | ? | + | + |
| ter Borg, Kelder (2017) | + | + | + | + | + | ? | ? | + | + |
| Retamozo et al. (2019) | + | + | + | + | + | + | + | + | + |
| Ramos-Casals et al. (1998) | + | - | - | + | - | ? | ? | + | + |
| Ramos-Casals et al. (2014) | - | + | + | - | + | ? | ? | + | + |
| Ramos-Casals (2015) | - | + | + | - | + | + | ? | + | + |
| Quartuccio et al. (2017) | - | - | - | - |  | ? | ? | + | + |
| Pertovaara et al. (2001) | - | - | - | - | + | - | + | + | + |
| Ioannidis et al. (2002) | - | - | + | - | + | ? | ? | + | + |
| Haga, Andersen, Pen (2011) | - | - | - | - | - | ? | ? | + | + |
| Gottenberg et al. (2013) | - | + | + | - | - | + | + | + | + |
| García-Carrasco et al. (2002a) | - | - | - | + | + | ? | ? | + | + |
| García-Carrasco et al. (2002b) | - | - | - | - | - | + | - | + | - |
| Flores-Chávez et al. (2018) | + | + | + | + | - | ? | ? | + | + |
| Ramos-Casals et al. (2004) | - | - | - | + | - | + | + | + | + |

+=Yes; -=No; ?=Unclear

**Hematological Manifestations**

| **Study ID** | **Was the sample frame appropriate to address the target**  **population?** | **Were study participants sampled in an appropriate way?** | **Was the sample size adequate?** | **Were the study subjects and the setting described in**  **detail?** | **Was the data analysis conducted with sufficient coverage**  **of the identified sample?** | **Were valid methods used for the identification of the**  **condition?** | **Was the condition measured in a standard, reliable way**  **for all participants?** | **Was there appropriate statistical analysis?** | **Was the response rate adequate, and if not, was the low**  **response rate managed appropriately?** |
| --- | --- | --- | --- | --- | --- | --- | --- | --- | --- |
| Vasilev et.al 1990 | + | + | + | + | NA | + | + | + | NA |
| Ramakrishna et.al (1992) | + | + | + | + | NA | + | + | + | NA |
| Tzioufas et al 1996 | + | + | + | + | NA | + | + | + | NA |
| Ramos Casal 1998 | + | + | + | + | NA | + | + | + | NA |
| Skoupoli et al 2000 | + | + | + | + | NA | + | + | + | NA |
| Aoki et al 2000 | + | + | + | + | NA | + | + | + | NA |
| Maria Garcia Carrasco et al 2002 | + | + | + | + | NA | + | + | + | NA |
| Vasilev et al 2004 | + | + | + | + | NA | + | + | + | NA |
| Theander et al 2004 | + | + | + | + | NA | + | + | + | NA |
| Alamanos et al 2006 | + | + | + | + | NA | + | + | + | NA |
| Friedman et al 2006 | + | + | + | + | NA | + | + | + | NA |
| RamosCasal et al 2008 | + | + | + | + | NA | + | + | + | NA |
| Barcelos et al 2009 | + | + | + | + | NA | + | + | + | NA |
| Baimpa et al 2009 | + | + | + | + | NA | + | + | + | NA |
| Brito-Zeron et al 2009 | + | + | + | + | NA | + | + | + | NA |
| Lin Dong-Fang et al 2010 | + | + | + | + | NA | + | + | + | NA |
| Zhou JG et al 2010 | + | + | + | + | NA | + | + | + | NA |
| Fauchais et al 2010 | + | + | + | + | NA | + | + | + | NA |
| Caramaschi et al 2012 | + | + | + | + | NA | + | + | + | NA |
| Malladi et al 2012 | + | + | + | + | NA | + | + | + | NA |
| Risselada et al 2013 | + | + | + | + | NA | + | + | + | NA |
| Baldini et al 2013 | + | + | + | + | NA | + | + | + | NA |
| Ramos Casal et al 2014 | + | + | + | + | NA | + | + | + | NA |
| Brito Zeron et al 2014 | + | + | + | + | NA | + | + | + | NA |
| Retamozo et al 2016 | + | + | + | + | NA | + | + | + | NA |
| Quartuccio et al 2017 | + | + | + | + | NA | + | + | + | NA |
| D Xu et al 2019 | + | + | + | + | NA | + | + | + | NA |

+=Yes; NA=Not Applicable
